# A Comprehensive Review of Variability in the Thermal Resistance (D-Values) of Food-Borne Pathogens—A Challenge for Thermal Validation Trials

**DOI:** 10.3390/foods11244117

**Published:** 2022-12-19

**Authors:** Aswathi Soni, Phil Bremer, Gale Brightwell

**Affiliations:** 1 Food System Integrity, Smart Foods and Bioproducts, AgResearch Ltd., Palmerston North 4414, New Zealand; 2 Department of Food Science, University of Otago, P.O. Box 56, Dunedin 9054, New Zealand; 3 New Zealand Food Safety Science and Research Centre, Palmerston North 4474, New Zealand

**Keywords:** D-values, statistical model, *Bacillus cereus*, *Cronobacter sakazakii*, *Escherichia coli*, *Listeria monocytogenes*, *Clostridium perfringens*

## Abstract

The thermal processing of food relies heavily on determining the right time and temperature regime required to inactivate bacterial contaminants to an acceptable limit. To design a thermal processing regime with an accurate time and temperature combination, the D-values of targeted microorganisms are either referred to or estimated. The D-value is the time required at a given temperature to reduce the bacterial population by 90%. The D-value can vary depending on various factors such as the food matrix, the bacterial strain, and the conditions it has previously been exposed to; the intrinsic properties of the food (moisture, water activity, fat content, and pH); the method used to expose the microorganism to the thermal treatment either at the laboratory or commercial scale; the approach used to estimate the number of survivors; and the statistical model used for the analysis of the data. This review focused on *Bacillus cereus*, *Cronobacter sakazakii*, *Escherichia coli*, *Listeria monocytogenes*, and *Clostridium perfringens* owing to their pathogenicity and the availability of publications on their thermal resistance. The literature indicates a significant variation in D-values reported for the same strain, and it is concluded that when designing thermal processing regimes, the impact of multiple factors on the D-values of a specific microorganism needs to be considered. Further, owing to the complexity of the interactions involved, the effectiveness of regimes derived laboratory data must be confirmed within industrial food processing settings.

## 1. Introduction 

Many food processing regimes use thermal treatment either as a standalone method or as one of the critical control steps to eliminate microbial contaminants and reduce enzyme activity. Thermal treatments are designed to ensure food safety and increase shelf life. The microbial safety of thermally processed food depends on the time and temperature regime used during processing. This could be equivalent to or comparable to pasteurization or sterilization. Pasteurization can be described as a moderate heat treatment of food at less than 100 °C, which aims to inactivate pathogenic vegetative microbial cells [1,2]. Thermal sterilization employs a heat treatment at 121 °C for up to 5 min, leading to the elimination of spores of *Bacillus* and *Clostridium* species [3,4]. The design of suitable thermal processing regimes relies upon several factors, including the type of media; the containers and apparatus used; and the intrinsic factors of the food such as the pH, moisture content, fat content, total solids, and water activity. The common objective of thermal regimes is to achieve at least a 6-log reduction in the number of the target bacterial species at the coldest spot in the food [5]. The regulations for pasteurization also depend on the food being treated and the target pathogen. For example, as per the Food and Drug Administration (FDA), a 6-log reduction in *Clostridium botulinum* (vegetative forms type E and nonproteolytic types B and F) is considered generally suitable for pasteurized seafood products [6], while at least a 5-log reduction in *Salmonella* is suitable for shelled eggs [7]. Hence, the thermal regime may also vary based on the product being produced and the level of inactivation required. To design effective thermal processing regimes, the thermal resistance of bacterial species is a key parameter that needs to be determined.

A term commonly used to address the thermal resistance of bacteria is the D-value, or decimal reduction time. The D-value of a given bacterial strain is defined as the time required at a specific temperature to achieve a 1-log (90%) reduction in number [8]. D-values play an important role in the computation of F-values for food processing. F-values, or minimum exposure times, are used to optimize thermal sterilization processes. F-values are defined as the treatment time required at a specific temperature, which are calculated on the basis of D-values to determine the lethal exposure at any temperature to eliminate the bacterial population to an acceptable limit [9]. To calculate the F-values, the D-values can be directly taken from the literature or, in the case of an unknown temperature, empirical models can be constructed with the D-values and temperature resistance coefficient Z (°C or °F) as parameters [9]. Therefore, D-values play an integral part in the design of the thermal treatment required by a given process to inactivate the food-borne pathogens and spoilage-related bacteria to an acceptable level. D-values are also used to compute the treatment required for the inactivation of enzymes; however, this is a separate topic and was not addressed in this review. 

## 2. Food-Borne Pathogens and Their Thermal Resistance

This review will aid in the understanding of the factors affecting the variation in thermal resistance reported for food-borne pathogens. For this purpose, *Bacillus cereus*, *Cronobacter sakazakii*, *Escherichia coli*, *Listeria monocytogenes*, and *Clostridium perfringens* were selected as pathogens of interest based on pathogenicity, the availability of publications on their thermal resistance, and their relevance in designing a method for the validation of any novel processing technology.

### 2.1. Bacillus cereus Spores

*B. cereus* is a spore-forming, Gram-positive aerobic or facultatively anaerobic member of the class Bacilli, order Bacillales, and family Bacillaceae. It is a motile, rod-shaped bacterium that is ubiquitous in the environment [10,11]. *B. cereus* is also part of a larger group known as “*B. cereus sensu lato (s.l.)*”, which consists of closely related bacterial species: *Bacillus anthracis*, *B. cereus sensu stricto* (s.s.), *Bacillus thuringiensis*, *Bacillus weihenstephanensis*, *Bacillus mycoides*, *Bacillus pseudomycoides, Bacillus cytotoxicus*, and *Bacillus toyonensis* [12]. The members of this group, including *B. cereus*, can produce spores, which help them to survive in adverse conditions such as heat, pH, and desiccation [13]. As a result, it is a challenge to eradicate these spores from processing lines, food contact surfaces, or even hard-to-reach areas in pipelines and utensils. Spores can germinate into vegetative cells under favorable conditions, such as (but not limited to) mild heat (<80 °C for up to 10 min); low pressure (<300 MPa); and the presence of germinants (alanine, lysine, etc.) [14,15,16]. *B. cereus* spores germinate into vegetative cells, which can produce two types of toxins, either emetic or diarrheal. Emetic food poisoning is a condition whereby the *B. cereus* cells produce a toxin called ceramide in the food, which leads to symptoms once the concentration exceeds 0.01 mg/g [3,17]. The symptoms of emetic food poisoning are vomiting within 5 to 6 h and nausea [17]. Diarrheal food poisoning caused by *B. cereus* is a result of the ingestion of the vegetative cells, which can subsequently multiply in the human intestine and produce three types of diarrheal enterotoxins (haemolysin, non-haemolytic three-component enterotoxin, and cytotoxin K) [11,12,17]. The symptoms include abdominal pain, watery diarrhea, and occasionally nausea within 24 h of the ingestion of the bacteria. *B. cereus* has been detected on various occasions in infant foods and dried milk products [18,19]; raw [20] and reheated rice [21,22]; meat and meat products [23]; and other food products, including spices, lentils, and vegetables [24]. 

The D_100_-values of *B. cereus* spores reported in model solutions such as buffers or water are listed in Table 1. The D-values (thermal resistance) of *B. cereus* spores at 100 °C reported in Table 1 range from 0.1 to 7.0 min. This variation could be attributed to several factors discussed in Section 3. 

The variation in D-values at a particular temperature for a specific *B. cereus* spore strain can be even greater when a solid food matrix is used as a heating medium (Table 2) as opposed to a liquid medium (Table 1). Table 2 includes a wide range of temperatures (80–100 °C) to address the variability of the data; however, a direct comparison cannot be made between the values reported in Table 1 and Table 2, as the strains, temperatures, matrix compositions, and setups differ. Additionally, most of the studies reported on the thermal resistance of *B. cereus* in food matrices (listed in Table 2) used temperatures below 100 °C.

While it is evident that the thermal resistance (D-values) can vary based on the matrix used and the strain assessed, there are no reports on the use of the same strain under different experimental conditions across two different laboratories. This makes it challenging to compare the actual differences based on any experimental artefacts or sporulation conditions.

### 2.2. Clostridium perfringens

*C. perfringens* is a spore-forming, Gram-positive, anaerobic, non-motile rod-shaped member of the class Clostridia, order Clostridiales, and family Clostridiaceae [32]. *C. perfringens* is a food-borne pathogen not only for humans but also for animals, and hence it is of concern for the pet food industry [33,34]. *C. perfringens* has been reported to produce 13 different types of toxins. Type A diarrhea and the less prevalent Type C human necrotic enteritis are considered to be food-borne diseases. The pathogenicity of *C. perfringens* in humans is related to either the formation of gas gangrene or to two types of foodborne diseases: the relatively mild, more commonly reported Type A diarrhea, and the less prevalent but more severe Type C human necrotic enteritis [32]. *C. perfringens* has been reported to cause enterotoxaemia in animals and necrotic enteritis in birds [32]. *C. perfringens* spores can survive in aerobic conditions and have been reported to be ubiquitous in nature, including in habitats such as soil, water, sewage, and the intestinal canal of humans and animals [32]. As a result, *C. perfringens* spores have been known to find their way into processing plants, where they are difficult to eliminate due to their resistance to heat and chemicals [35,36]. *C. perfringens* has been reported to form biofilms, which structurally protect the cells from oxygen as well as disinfectants such as hydrogen peroxide and quaternary ammonium chloride solutions [36]. *C. perfringens* spores have been detected in retail foods such as meat and meat products, dairy products, honey, vegetables, and fruits and vegetables in canned form [37,38]. Despite many foods receiving thermal treatment during processing, the presence of *C. perfringens* in these products indicates that some strains might have higher thermal resistance than the time–temperature regimes of the processing methods. The D-values of *C. perfringens* reported in the literature are listed in Table 3. 

Only a limited number of *C. perfringens* strains have been investigated for their D-values in food matrices; however, significant variation among the strains has been observed. A study by Wen and McClane [39] reported that *C. perfringens* spores carrying the chromosomal *cpe* (*C. perfringens* enterotoxin) gene demonstrated higher D-values (>90 min at 100 °C) than those carrying a plasmid-borne *cpe* gene. These findings suggested that apart from strain-based variation, matrix-based variation, and variation due to the experimental setup, the genes present and the location of the genes also have a potential effect. 

### 2.3. Shiga Toxin-Producing Escherichia coli (STEC)

*E. coli* is a Gram-negative, non-spore-forming, facultative anaerobe that belongs to the family Enterobacteriaceae, which is known to co-exist with other bacteria in the large intestines of human hosts. Depending on the strain, *E. coli* can either exist in a symbiotic relationship with the human host by providing resistance against pathogenic organisms, or it can be pathogenic, causing intestinal diseases [42]. Shiga toxin-producing *E. coli* (STEC) is one of the four recognized groups of *E. coli* that are known to cause diarrhea in humans. They are either food- or water-borne, especially after cross-contamination via the fecal–oral route [43,44,45]. A study by Scallan et al. [46] reported that approximately 175,000 food-borne infections per year in the United States are caused by STEC. *E. coli* is not known to form endospores; hence, this microorganism is not as resistant to thermal processing or disinfection regimes as *Bacillus* or *Clostridium* spores. However, it has been reported to be a persistent contaminant for various reasons. The thermal resistance of *E. coli* has been reported to be variable, with some strains exhibiting a stable and thermotolerant phenotype, while for other strains certain environmental challenges, such as a sudden increase in cultivation temperature, have been associated with the evolution of thermotolerance [47,48]. Due to the severity of the disease they cause and the variability associated with reported bio-adaptable, evolving thermal resistance, *E. coli* remain a challenge for the food industry. The thermal resistance (D-values) of *E. coli* in different types of media/matrices are listed in Table 4. 

### 2.4. Cronobacter sakazakii

*C. sakazakii* is a motile, Gram-negative, non-spore-forming, rod-shaped opportunistic food-borne pathogen, formerly known as *Enterobacter sakazakii* [52]. *C. sakazakii* belongs to the genus Cronobacter within the family Enterobacteriaceae [52,53]. *C. sakazakii* has gained significant attention from food safety experts due to its infrequent association with neonatal illness following the consumption of contaminated infant formula. The symptoms of infection include necrotizing enterocolitis, bacteremia, and meningitis in infants [54,55] and immunocompromised adults [56,57]. *C. sakazakii* has emerged as a food safety challenge due to its tolerance to desiccation and antibiotics [58]; its ability to form biofilms [59]; and its evolving resistance as a stress response mechanism to various non-thermal and thermal food processing methods [60], which could help to explain its ability to contaminate and survive in powder infant formula [61,62]. Consequently, in this review, we discussed this pathogen with milk as the main food matrix (Table 5).

*Cronobacter* strains have been reported to not survive either commercial pasteurization treatment (>70 °C) or a reconstitution process that uses hot water (> or =70 °C) [64]. However, their D-values can significantly differ based on the matrix used, for example, whole milk or low-fat milk, as reported by Osaili et al. [64]. Additionally, the culture conditions; experimental conditions; and, most importantly, commercial versus laboratory setups could also cause variation.

### 2.5. Listeria monocytogenes

*Listeria monocytogenes* is a Gram-positive, facultative intracellular rod-shaped bacteria from the family Listeriaceae and the order Bacillales [67]. L. monocytogenes is a food-borne pathogen that causes listeriosis, a disease with high hospitalization and fatality rates and increased effects on immunocompromised individuals, newborn babies, and pregnant women [68,69]. Listeriosis presents either with common symptoms such as fever and diarrhea or as invasive listeriosis in rare cases when the pathogen exploits actin polymerization for intracellular movement, thereby spreading to a vast range of host tissues and organs (such as the liver), leading to symptoms including liver cirrhosis, meningitis, and septicemia [68,70]. In the case of pregnant women, invasive listeriosis can lead to miscarriage, stillbirth, premature delivery, or life-threatening infections in the newborn [71,72].

*L. monocytogenes* has been periodically detected in a wide range of raw and processed food products, such as green leafy salads and ready-to-eat meals [73], meat and meat products [74], and dairy products [75,76]. While the bacteria cannot survive sterilization, antimicrobial resistance has been reported against chemicals used in disinfection [77] as well as various degrees of tolerance to mild/moderate thermal regimes [78,79]. The variation in the thermal resistance of L. monocytogenes (Table 6) is a concern for food manufacturers. 

The *L. monocytogenes* Scott A strain was originally found to have caused a listeriosis outbreak in 1983 in Massachusetts [85]. This strain has been widely used for validation trials in thermal and non-thermal food processing studies. Among the numerous factors that affect the thermal resistance of *L. monocytogenes* Scott A, the food matrices have a significant influence. For example, the D-values in meat and TSB varied by at least 2 min (Table 1), although the laboratory conditions might have had an influence on the results. 

### 2.6. Surrogates for Food-Borne Pathogens

Food processing industries can benefit from novel technologies that use mild to moderate heat and hurdle or synergic approaches to achieve bacterial elimination (safety) while preserving heat-sensitive nutrients [86]. However, before commercialization, any novel technique needs to be validated using various approaches including challenge testing trials with the bacterial contaminants of concern to demonstrate and document targeted efficacy [87]. Validation methods often rely on surrogates of bacterial pathogens because of safety and sanitary concerns. Surrogate bacterial strains are selected based on their genetic similarity to the target pathogens, non-pathogenic nature, similar level of susceptibility to processing (thermal or non-thermal), and feasibility of use in laboratory and processing premises [88]. Some regularly used surrogates for process validation are *E. coli* (non-STEC), *Listeria innocua* for *L. monocytogenes*, *B. subtilis* for *B. anthracis* and *B. cereus*, non-pathogenic strains of *Salmonella* spp. for pathogenic strains, and *C. sporogenes* for *C. botulinum*, as reviewed in detail by Hu and Gurtler [88]. The challenge associated with using an appropriate surrogate for any study is the lack of validation of their similarity to the pathogenic species of interest. This includes the similarity in inactivation kinetics, structural damage (sublethal or lethal), growth parameters, or survivability under certain conditions alongside a statistical verification of similarity. The thermal resistance of bacterial surrogates must be well understood before designing any challenge studies, especially in the target food matrix (not a model buffer-based medium). However, this topic is worthy of its own review due to the large amount of relevant data, so it is not discussed further in the current review.

## 3. Factors Affecting Variation in D-Values Reported in the Literature 

Thermal resistance as monitored by the D-value remains the key parameter used to design time–temperature regimes for thermal processing. However, D-values can be highly variable due to various factors. These include the type of strain being treated, the methods used to estimate the D-values, and the conditions prevailing in the laboratory as compared to those in the food processing industry. Each of these parameters is explained in the following sections based on the reported values for different bacterial pathogens listed in Table 1, Table 2, Table 3, Table 4, Table 5 and Table 6. 

### 3.1. Laboratory Scale versus Commercial Applications

In a laboratory, the isothermal conditions for the desired temperature are usually achieved using either a water bath [64,81,83], a thermoresistometer [66], or an oil bath [41]. An oil bath is preferred if the desired temperature is above 95 °C, to prevent evaporation and hence temperature imbalance. In comparison, at a commercial scale, a regular trial to evaluate thermal resistance is carried out in a pasteurization unit or a sterilization unit (e.g., retort) for temperatures above 100 °C. Commercial processes are frequently continuous and involve larger flow rates and volumes, whereas laboratory-scale trials are frequently carried out with small volumes and samples in a batch process, which also creates a difference in the volume being treated in both cases. Another key issue with any thermal resistance trial is the heat transfer time and the come-up time, which can be defined as the time required for the center of the food being treated to match the preset temperature of the oil bath or water bath (preferred heating medium). While the heat transfer can be monitored and controlled well in a laboratory-based setup, the inactivation curve can either keep the trial isothermal, i.e., the first sampling point is taken after the desired temperature has been achieved, or non-isothermal, i.e., the part of the curve including the come-up time is also included in the sampling. Although D-values are usually obtained as the inverse of the slope, and hence, theoretically, the linear portion is used for this computation, there could be a shoulder in the graph depending on whether the sampling time points were before or after the come-up time was obtained, which in turn might affect the time required for the initial log reduction. Based on the volume of food being treated, the come-up times in a lab-based setting will be different to those of commercial processes. Therefore, the total time required for the complete inactivation may differ. In addition, it is challenging to reproduce industrial conditions using laboratory-scale apparatus. Therefore, there is concern that D-values obtained under laboratory conditions designed to match commercial conditions could differ significantly from the actual values achieved under commercial conditions. The reason for such differences is believed to be due the matrix used, the volume involved, the heating rates, and the level of agitation or turbulence involved.

Heating rates have been reported to have a significant effect on bacterial inactivation. In general, D-values are determined while using heating rates of 1 to 20 °C /min; however, higher heating rates of up to 50 °C/min (using a heat exchanger and thermoresistometer) were capable of delivering better inactivation than the predicted values using lower heat exchange, and the difference was more than 6-log cycles in the same amount of time [89]. The heating rate, for example, can have a significant effect on the thermal resistance of *E. coli*, with high heating rates resulting in an additional sensitization to heat [89]. Industrial pasteurization units are generally designed to have a fast heating rate, which allows them to achieve a better reduction in bacterial numbers than that obtained using laboratory-scale oil or water baths.

### 3.2. Role of Methods in Variation When Estimating Thermal Resistance of Bacteria in Food

While the overall steps in the studies designed to estimate D-values are similar, the setup, containers, heating medium and apparatus, recovery media, and methods can vary significantly between laboratories. For example, the containers used to hold liquid or solid samples for the thermal inactivation experiments include capillary tubes, vials, test tubes, plastic sealed bags, flasks, and bottles (Table 1, Table 2, Table 3, Table 4, Table 5 and Table 6). The equipment used for heating can also be variable, including water baths, oil baths, thermal plates, and thermoresistometers. Additionally, the use of various types of media to recover and enumerate the surviving bacteria can be highly inconsistent between trials—specifically, when thermal treatments are known to cause sublethal (repairable) injury if the temperature and time combination is below that required for complete lethality. To prevent any contamination from other bacteria or in cases where a cocktail of multiple strains is being assessed, selective media plating is extensively used to recover the survivors of thermal treatment. However, it has been reported that sublethally injured cells can fail to grow on selective media plates [90]. This is because of the use of antibiotics, sodium-based components, and any osmoregulators, which can further damage the sublethally injured cells, whose structural integrity is known to be compromised [91,92]. If sublethally injured cells are not recovered and, consequently, not enumerated, this can lead to the false assumption of reduction in numbers by the heat treatment alone. When the treatment matrix is food and not a model fluid, the recovery of the bacterial survivors adds another layer of complexity due the interaction between the bacteria or spores and the food components. In commercial premises, thermal trials are usually conducted in food matrices, whereas laboratory trials frequently use model systems. However, the variation in D-values obtained owing to the matrix effect is high enough to restrict the direct application of these results in practice, and, in principle, every food product needs a separate evaluation of the target strain’s decimal reduction time when developing a thermal regime for inactivation. 

Another major factor that can impact on the thermal resistance of bacterial cells or spores is the number of cells in the initial inoculum. To obtain an acceptable survival curve, it is generally decided to start the trial with a high inoculum level (10^7−8^ CFU/unit); however, this is usually not the situation in commercial food prior to thermal treatment, and this may also be a cause of variation. In a larger population, the reduction in bacterial numbers is significantly evident only after a certain time, known as the ‘lag time’ or “shoulder” [93,94]. This is especially applicable to a population of highly heat-resistant bacteria (spore-formers), where a similar strain might have subpopulations that differ in thermal resistance [95]. It could also be related to the presence of agglomerates in the spore suspension and any damage that might have occurred to the spores after treatment but before enumeration [96]. Therefore, the theoretical linear form of reduction is not seen. 

### 3.3. Composition, Conditions, and Type of Food Matrix

The effect of the food matrix on bacterial inactivation and hence thermal resistance can be considered with regards to its pH; water activity; fat composition; salt composition; and physical state (solid, liquid, or semi-solid). Huertas, Álvarez-Ordóñez, Morrissey, Ros-Chumillas, Esteban, Maté, Palop, and Hill [66] investigated the effect of different reconstitution and handling scenarios on the thermal resistance of *C. sakazakii* in a commercial brand of infant formula. D-values were first determined in the model system and milk (Table 5), followed by a study to understand the effect of infant formula reconstitution with water at different temperatures (50–70 °C) on bacterial inactivation. It was clear that the use of water at temperatures between 50 and 65 °C for reconstitution did not cause a significant inactivation of *C. sakazakii* cells; however, reconstitution at 70 °C reduced the bacterium to a level equivalent to 5-log CFU/mL. The use of higher temperatures reduced the protective effect of the food matrix on *C. sakazakii.*

The effect of moisture on food safety is of particular concern to food regulators due to the ability of various food-borne pathogens to survive in low-moisture food products. For example, *Salmonella* spp. has been known to survive in spices, dry nuts, chocolate, and peanut butter; *B. cereus* spores have been found in rice; and *C. botulinum* spores have been detected in honey [97,98]. Low-moisture foods have lower heat transfer rates due to their lower water mobility, which in turn reduces the uniform distribution of heat in the food being treated, which can generate cold spots or volumetric centers that receive less heat compared to the outer regions of the food. This leads to the survival of thermally resistant spores or bacteria in low-moisture foods. A study by Alderton et al. [99] estimated the D-values of *C. botulinum* 62 A at different levels of water activity (0.1–0.9). Spores were suspended in either calcium acetate (to increase their thermal resistance) or in water post-treatment with 0.1 N HCL to reduce the thermal resistance [99,100]. This experiment aimed to understand the effect of water activity on the thermal resistance of these spores under conditions whereby they were conferred with additional thermal resistance or reduced thermal resistance. The results indicated the significant effect of water activity on D-values and Z-values, which are defined as the temperature increment needed for a ten-fold reduction in the D-values [101]. While it was evident that lower water activity led to higher resistance in all three types of spores being tested, the Z-values were a better indication of the overall effect, with a reduction of up to 1–15 °C observed after increasing the water activity from 0.0 to 0.9 [99]. Therefore, the water activity of different food products could be related to the thermal resistance and survival of bacterial pathogens, especially spore-formers. 

Another factor that has a strong influence on the thermal resistance of bacteria in food is the pH. Acidic pH values are known to reduce the thermal resistance of *C. sporogenes* [102], *Geobacillus stearothermophilus* spores [103], *C. botulinum* 213B [104], and *L. monocytogenes* [105]. In contrast, regular exposure to a low pH or acid stress can confer resistance to certain bacterial cells, as reported for *C. sakazakii* [106]. While the mechanism of action was not studied, it was postulated that the sigma factor σ2, 2-component signal transduction systems (e.g., PhoP and PhpQ) or the major iron and regulatory protein Fur and alternative sigma factor σ2, encoded by *rpoS*, could be responsible, as earlier reported in the case of *Salmonella* and *Shigella* spp. [106,107,108]. Similarly, pH-dependent acid resistance in *E. coli* O157:H7 has been reported to confer cross-protection and therefore increase D-values [109]. The D_58_-values reduced from 5.4 min to 2.1 min when the medium was acidified TSB (pH 4.7) and non-acidified TSB (pH 7.2), respectively [109]. However, it is difficult to point to acid tolerance, acid sensitivity, and the effect of water activity or pH as the single or primary reason when the treatment matrix is food. Additionally, for a reduction in pH, additional components/solutions need to be employed, which in turn change the composition of food and can also have an impact on the physical state and thermal resistance of bacterial cells in vegetative form or spores. It can therefore be concluded that in any food matrix, there could be more than one factor affecting the predicted thermal resistance of a specific bacterial strain. 

### 3.4. Statistical Models Used in Prediction/Estimation

Predictive models use thermal death data (D-values) from the literature in combination with microbiology, mathematics, and statistics to design mathematical models that can predict thermal resistance based on the existing variables provided, such as the matrix and bacterial strain. Several statistical models have been reported in the literature to be useful for understanding bacterial thermal resistance. A few such models, their applications, and some of their features are listed in Table 7.

From the models reported in Table 7, it can be observed that not all survival curves plotted using logarithmic reduction in the bacterial population against the time of exposure at a pre-set and controlled temperature are necessarily linear. The survival curves may include a tail due to bacterial populations with variable thermal resistance that was either conferred or pre-acquired. The survival curves can also be sigmoidal, with a peak in the center and slow logs at both ends, or can have an extensive tail that could significantly alter the D-values. Hence, more than one model might be useful for studies estimating or predicting the survival of bacteria. 

### 3.5. Inherent Resistance in Subpopulations of Bacterial Species

Bacterial spore-formers such as *B. cereus* and *C. sporogenes* are used as bioindicators of sterility and are often reported to produce tails in survival curves [116,117,118]. While there are several models and explanations for this, the most common reason is believed to be the existence of super-dormant spores in the population. Super-dormant is a term used for a subpopulation of spores that exhibit a significant difference in their ability to germinate, as well as having a higher thermal resistance than their counterparts in the population [119,120,121]. While the exact mechanism behind this elevated resistance in super-dormant spores is not known, one postulated reason is the difference in the number of receptors in the intermembrane of these spores, which makes them less responsive to the external environment, including wet heat treatments. A study by Zhuang et al. [122] indicated that a specific group of *B. cereus* spores encoding the *panC* gene had comparatively higher D-values. The *panC* gene is a housekeeping gene that encodes pantothenate-β-alanine ligase and is often used to identify and classify *B. cereus* subtypes with specific phenotypes, including high wet heat resistance. A study of factors contributing to the thermal resistance in *Clostridium perfringens* indicated the presence of the plasmid *cpe* gene [40], which codes for *C. perfringens* enterotoxin and is associated with food poisoning [35,40].

In non-spore-forming bacteria, similarly extended tailing, upward concavity in survival curves, and elevated resistance have been observed in the case of *Cronobacter* spp., and the genes associated with this are *rimP*, coding for the ribosome maturation protein RimP, and *ompL,* coding for outer-membrane porin L (OmpL) [66]. *Cronobacter* spp. has been known to exhibit strains with a thermotolerance DNA region that have been reported to show at least two times higher thermal resistance than the strains without this DNA region [123]. The thermotolerant region in *C. sakazakii* ATCC 29544 is 8 kbp with 22 open reading frames and consists of a cluster of conserved genes, including stress response genes for heat, oxidation, and acid [123]. A similar thermotolerant region of DNA has also been identified in *E. coli* KL 53 [123,124,125]. Thermal resistance in some *E. coli* strains (including STEC) is reported to be associated with the increased structural integrity of the cell envelope, the expression of heat shock proteins, and chaperone synthesis [126]. *L. monocytogenes* are considered to be highly heat-resistant non-spore-forming bacteria. The heat resistance is reported to be mediated through a plasmid-borne gene called ATP-dependent protease *cpL*, which encodes for ATP-dependent protease ClpL [127]. However, the exact mechanism of action and pathway affected are not clear. Another study indicated the effect of lactic acid stress on cross-protection against heat in *L. monocytogenes* [128]. The genes responsible for the heat stress were related to heat shock and chaperons (*ctsR*, *mcsB*, *clpP*, *clpB*, and *clpE*) [128,129]. 

Therefore, the presence of specific subpopulations within different strains that have an enhanced heat tolerance and therefore higher D-values contributes to the occurrence of nonlinear survival curves. Although statistical models (Table 7), are useful to predict lethality values, it can be concluded that further studies to understand the mechanism of induced thermal tolerance as part of cross-protection in food processing are necessary. 

## 4. Research Gaps and Conclusions

Recent developments in novel approaches to eliminate food-borne pathogens and spoilage-related bacterial contaminants from food have led to an increase in validation work. While most of the novel technologies aim to use mild-to-moderate heat, increased knowledge about the thermal resistance of either bioindicators or any pathogen of interest is the first step to designing a processing regime. For this work, the existing literature is referred to; however, there are research gaps that need attention to ensure benefits for food processors and regulatory bodies. Some of the research gaps identified by this review were:

1. A lack of information on the come-up time (CUT) and models that could integrate this time into account.
2. The limited database of D-values of food-borne pathogens such as *B. cereus*, *C. botulinum*, *E. coli* (STEC), *C. sakazakii*, and *L. monocytogenes* in different types of matrices, including buffers and food matrices (meat, milk, dried products) under commercial and laboratory-based setups.
3. Only a few reports indicate correlations between industrial equipment (for example, pasteurization units and retorts) and laboratory-scale equipment used for the determination of D-values. Consensus with the statistical models used in laboratory-based setups would benefit from validation or application in commercial setups.
4. The factors influencing the formation of shoulder-like survival curves and extended tails in survival curves require further investigation.

It can therefore be concluded that while understanding the effect of a single factor on D-values is useful, care should be taken not to directly apply such findings to industrial-scale processing when the D-values of bacterial contaminants in the food being treated might not be limited by one factor. The microenvironments of each food type should be considered, and understanding how they influence the thermal resistance of specific bacterial species will contribute towards overcoming this challenge. Before designing a thermal process to extend shelf life and enhance safety, it is essential to individually evaluate the combined effect of the bacterial species being targeted along with the food matrix being used on the D-values. Most of the data available on the D-values of microorganisms (non-spore-formers and spore-formers) are based on experiments/trials using a model system but not food (buffer or water) as the matrix. Therefore, these values could be significantly different from the real scenario in a food-based matrix. While such an experiment could be an initial reference point, it should not be considered as a final reference for estimating the time required for heat treatment in a food-based matrix. Owing to the variation in D-values reported in the literature for the same strain, it is concluded that when designing thermal processing regimes, the impact of multiple factors on the D-values of a specific microorganism needs to be considered. It is therefore recommended to conduct D-value estimations in the food matrix while simulating the commercial conditions, followed by confirmatory trials within industrial food processing settings.

## Figures and Tables

**Table 1:** Summary of D_100_-values (thermal resistance) for *B. cereus* spores along with the medium and the method used.

*Bacterial* spp. and Strain	D-Value (min)	Matrix Used	Method Used	Reference
*B. cereus* 4342	0.42–5.03	McIlvaine buffer	Thermoresistometer	[25]
*B. cereus* 7004	0.06–0.12	McIlvaine buffer	Thermoresistometer	[25]
*B. cereus* 9818	1.2–10.9	McIlvaine buffer	Thermoresistometer	[25]
*B. cereus* ADQP 407	1.04–5.57	Tryptone salt broth	Capillary tubes and thermostat	[26]
*B. cereus ATCC 14579-5*	0.9	Phosphate buffer (0.067 M)	Capillary tubes and thermostat	[26]
*B. cereus* R_96_	6.9	Phosphate buffer (0.067 M)	Capillary tubes and thermostat	[26]
*B. cereus* B_4_ac	2.2	Phosphate buffer (0.067 M)	Capillary tubes and thermostat	[26]
*B. cereus* B_4_ac	1.6	De-ionized water	Capillary tubes and thermostat	[26]
*B. cereus* B_6_ac	1.7	Phosphate buffer (0.067 M)	Capillary tubes and thermostat	[26]
*B. cereus* B_6_ac	1.4	De-ionized water	Capillary tubes and thermostat	[26]
*B. cereus* 4810/72	4.4	Phosphate buffer (0.067 M)	Capillary tubes and thermostat	[26]
*B. cereus IP5832*	1.8	Infant milk formula (total solids: 10%)	Metal tubes in a shear field using a rheometer	[27]
*B. cereus IP5832*	3.5	Infant milk formula (total solids: 50%)	Metal tubes in a shear field using a rheometer	[27]

**Table 2:** D-values (thermal resistance) for *B. cereus* spores and the food matrix used.

*Bacterial* spp. and Strain	D-Value (min)	Matrix Used	Method Used	Reference
*B. cereus IP5832*	D_100_ = 1.8	Infant milk formula (total solids: 10%)	Metal tubes in a shear field using a rheometer	[27]
*B. cereus IP5832*	D_100_ = 3.5	Infant milk formula (total solids: 50%)	Metal tubes in a shear field using a rheometer	[27]
*B. cereus* DSM 4313 and DSM 626 (cocktail)	D_95_ = 2.0 min	Blended luncheon meat	Sterile polyethylene bags in a water bath	[28]
*B. cereus NZRM 984*	D_70_ = 8.6	Skim milk	Vacuum-packed food-grade sterile pouches in a water bath	[29]
*B. cereus NZRM 984*	D_70_ = 2.3	Beef slurry	Vacuum-packed food-grade sterile pouches in a water bath	[29]
*B. cereus* CM 2275	D_100_ = 1.92	Carrot juice (pH 6.2)	Capillary tubes and water bath	[30]
*B. cereus* ATCC 14579-8	D_97.5_ = 3.7	Rice broth	Steel containers via standard cooking	[31]

**Table 3:** Summary of D-values (thermal resistance) for *C. perfringens* spores along with the matrix used.

*Bacterial* spp. and Strain	D-Value (min)	Matrix Used	Method Used	Reference
*C. perfringens* cocktail (DSM 11784, NCTC 10614, and NCTC 08237)	D_100°C_ = 2.2	Blended luncheon meat	Sterile polyethylene bags in a water bath	[32]
*C. perfringens* type ANCTC8239	D_100°C_ = 93	Duncan–Strong (DS) sporulation medium	Batch thermal treatment with regular sampling	[39]
*C. perfringens* 8239	D_100°C_ = 124	Sterile distilled water	Batch thermal treatment with regular sampling	[40]
*C. perfringens* type A8798	D_104°C_ = 2.9	Phosphate buffer 0.067 M (pH 7.0)	Borosilicate glass tubes and oil bath	[41]
*C. perfringens* type A8798	D_104°C_ = 6.1	Commercial beef gravy	Borosilicate glass tubes and oil bath	[41]

**Table 4:** Summary of D-values (thermal resistance) for *E. coli* (STEC) and non-STEC strains in the literature along with the matrix used.

*Bacterial* Species and Strain	D-Value (min)	Matrix Used	Method Used	Reference
*E. coli* O26 STEC	D_60°C_ = 0.51	Apple juice	Thermostatically controlled water bath using glass vials	[49]
*E. coli* O45 STEC	D_60°C_ = 0.66	Apple juice	Thermostatically controlled water bath using glass vials	[49]
*E. coli O157:H7* STEC	D_60°C_ > 5.0	Luria–Bertani broth	Eppendorf tubes in dry bath incubator	[48]
Cocktail of 5 *E. coli* strains (STEC and non-STEC)	D_60°C_ = 0·1 to 0·5	Luria–Bertani broth	Eppendorf tubes in dry bath incubator	[50]
*E. coli* K12	D_60°C_ = 0.22	Liquid egg	Aluminum-based thermal-death-time disk in a water bath	[51]

**Table 5:** Summary of D-values (thermal resistance) for *C. sakazakii* along with the matrix used.

*Bacterial* spp. and Strain	D-Value (min)	Matrix Used	Method Used	Reference
*Enterobacter sakazakii*	D_60°C_ = 2.5	Reconstituted dried infant formula	Stainless-steel flat-bottomed centrifuge tubes in a water bath	[63]
*C. sakazakii* strains isolated from milk (n = 4)	D_58°C_ = 0.66	Lactose-free infant milk formula	100 mL capacity Duran bottles in a water bath with regular sampling	[64]
*C. sakazakii* strains isolated from milk (n = 4)	D_58°C_ = 0.51	Skim milk	100 mL capacity Duran bottles in a water bath with regular sampling	[64]
*C. sakazakii* strains isolated from milk (n = 4)	D_58°C_ = 0.68	Whole milk	100 mL capacity Duran bottles in a water bath with regular sampling	[64]
*E. sakazakii NCTC* 11467	D_60°C_ = 1.24	Saline solution	Glass test tubes (with stirrer) in a water bath	[65]
*E. sakazakii NCTC* 11467	D_60°C_ = 2.78	Rehydrated infant formula	Glass test tubes (with stirrer) in the water bath	[65]
*C. sakazakii* DPC 6529	D_58°C_ = 0.6	LB broth	Thermoresistometer Mastia vessel	[66]
*C. sakazakii* DPC 6529	D_58°C_ = 0.6	Infant formula	Thermoresistometer Mastia vessel	[66]

**Table 6:** Summary of D-values (thermal resistance) for *L. monocytogenes* along with the matrix used.

*Bacterial* spp. and Strain (Where Reported)	D-Value (min)	Matrix Used	Method Used	Reference
*L. monocytogenes* Scott A	D_61°C_ = 2.04	Meat (low-fat turkey)	Meat slices sealed in vacuum packs in a water bath	[80]
*L. monocytogenes* Scott A	D_62°C_ = 1.2	Fatty (30.5% fat) ground beef	Glass thermal death time tubes in circulating water bath	[81]
*L. monocytogenes* Scott A	D_62°C_ = 0.6	Lean (2.0% fat) ground beef	Glass thermal death time tubes in circulating water bath	[81]
*L. monocytogenes*	D_62.5°C_ = 2.23	Ground duck Muscle meat	Gas/moisture barrier (plastic) bag in a water bath	[82]
*L. monocytogenes* 1151	D_60°C_ = 0.87	Tryptic soy broth (TSB)	Capillary tubes in a water bath	[83]
*L. monocytogenes* Scott A	D_60°C_ = 0.34	TSB	Capillary tubes in a water bath	[83]
*L. monocytogenes* Scott A	D_60°C_ = 0.58	Brain heart infusion (BHI) broth	250 mL flasks in a water bath with regular sampling	[84]
*L. monocytogenes* L6	D_60°C_ = 4.1	Brain heart infusion (BHI) broth	250 mL flasks in a water bath with regular sampling	[84]

**Table 7:** Statistical models for D-value estimation and prediction.

Model	Details	References
Weibull distribution	Takes biological variation into account Accounts for the nonlinearity of semilogarithmic survival curves	[110]
Bigelow model	Considers the first-order equation Does not account for nonlinear curves/shoulders.	[111]
Log-Logistic Model	Assumes that bacterial cells in a population can have non-identical heat resistance Assumes that the difference in the thermal resistance in a given population is permanent Accounts for sigmoidal microbial inactivation data	[112]
Vitalistic model	Assumes that a subpopulation in the bacterial population under treatment is highly resistant to heat/inactivation treatment Covers either lags or tailing attributed to the tail/shoulder	[113]
Quasi-chemical model for microorganism growth–death	Combines the concepts of chemical kinetics and microbial growth in foods Uses functions such as functions of aw, pH, and temperature Considers negative feedback mechanisms such as nutrient depletion and secondary byproduct accumulation	[114]
Gompertz model	Takes into consideration the initial microbial load, lower quantification limit, and come-up time (CUT) Considers nonlinear sigmoidal curves Effective in modeling both linear survival curves and non-linear curves containing a shoulder and tail	[115]

## Data Availability

Data is contained within the article.
